# 2-(Methoxy­meth­oxy)-1-(4-oxobicyclo­[3.1.0]hexan-1-yl)ethyl 4-nitro­benzoate

**DOI:** 10.1107/S160053680706686X

**Published:** 2007-12-21

**Authors:** Jing Li, Todd L. Lowary, Michael J. Ferguson

**Affiliations:** a Alberta Ingenuity Centre for Carbohydrate Science, Department of Chemistry, University of Alberta, Edmonton, AB, T6G 2G2, Canada; b X-ray Crystallography Laboratory, Department of Chemistry, University of Alberta, Edmonton, AB, T6G 2G2, Canada

## Abstract

In the title compound, C_17_H_19_NO_7_, the cyclo­pentane ring is in an envelope conformation in which the methyl­ene group forming the flap is *cis* to the cyclo­propane group. The relative configuration between the 4-nitro­benzo­yloxy substituent on the side chain and the cyclo­propane ring is *trans* and the methoxy­lmethyl group adopts the expected conformation in which the two O atoms are *gauche* to one another.

## Related literature

For the synthesis of mimetics of biologically important furan­oside rings, see: Callam & Lowary (2000 ▶, 2001 ▶); Callam *et al.* (2001 ▶); Centrone & Lowary (2002 ▶). For examples of crystal structures of bicyclo­[3.1.0]hexane systems, see; Gurskaya *et al.* (1990 ▶, 1996 ▶); Gallucci *et al.* (2000 ▶); Garcia *et al.* (1992 ▶); Guthrie *et al.* (1981 ▶); Màrton-Merész *et al.* (1983 ▶); Biswas *et al.* (1996 ▶); Bai *et al.* (2004 ▶). For related literature, see: Hamon & Shirley (1988 ▶); Li & Lowary (2008 ▶); Wolfe (1972 ▶).
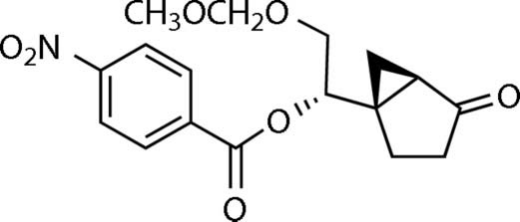


## Experimental

### 

#### Crystal data

C_17_H_19_NO_7_
*M* *_r_* = 349.33
Triclinic, 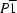

*a* = 8.3387 (5) Å
*b* = 10.1389 (6) Å
*c* = 10.4935 (6) Å
α = 98.5259 (8)°
β = 100.4967 (8)°
γ = 101.1562 (7)°
*V* = 840.22 (9) Å^3^
*Z* = 2
Mo *K*α radiation
μ = 0.11 mm^−1^
*T* = 193 (2) K
0.52 × 0.50 × 0.47 mm

#### Data collection

Bruker PLATFORM diffractometer SMART 1000 CCD area-detector
Absorption correction: multi-scan (*SADABS*; Sheldrick, 2003 ▶) *T*_min_ = 0.946, *T*_max_ = 0.951
7436 measured reflections
3825 independent reflections
3476 reflections with *I* > 2σ(*I*)
*R*_int_ = 0.009

#### Refinement

*R*[*F* ^2^ > 2σ(*F* ^2^)] = 0.042
*wR*(*F* ^2^) = 0.120
*S* = 1.05
3825 reflections
227 parameters
H-atom parameters constrained
Δρ_max_ = 0.31 e Å^−3^
Δρ_min_ = −0.17 e Å^−3^

### 

Data collection: *SMART* (Bruker, 1997 ▶); cell refinement: *SAINT* (Bruker, 1997 ▶); data reduction: *SAINT*; program(s) used to solve structure: *SHELXS97* (Sheldrick, 1990 ▶); program(s) used to refine structure: *SHELXL97* (Sheldrick, 1997*a* ▶); molecular graphics: *SHELXTL* (Sheldrick, 1997*b* ▶); software used to prepare material for publication: *SHELXTL*.

## Supplementary Material

Crystal structure: contains datablocks I, global. DOI: 10.1107/S160053680706686X/lh2582sup1.cif

Structure factors: contains datablocks I. DOI: 10.1107/S160053680706686X/lh2582Isup2.hkl

Additional supplementary materials:  crystallographic information; 3D view; checkCIF report

## Figures and Tables

**Fig. 1.**
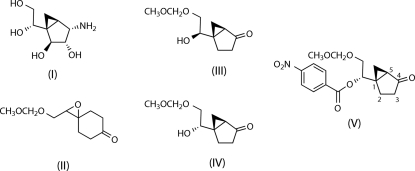
Schemes of title (IV) and related compounds.

**Fig. 2.**
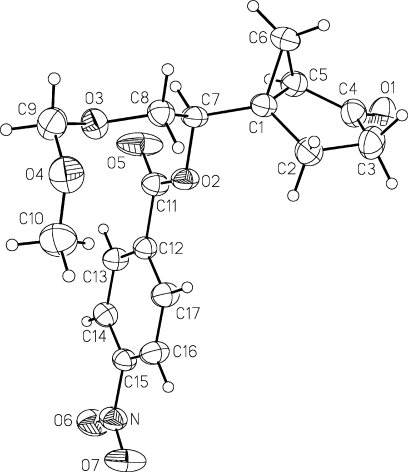
Perspective view of (V), showing the atom labelling scheme. Non- hydrogen atoms are represented by ellipsoids at the 50% probability level. Hydrogen atoms are shown with arbitrarily small radii.

## supplementary crystallographic information

### Comment

In the course of our studies on the synthesis of mimetics of biologically important furanoside rings (Callam & Lowary, 2000; Callam & Lowary, 2001; Callam *et al.*, 2001; Centrone & Lowary, 2002), we targeted compounds of the general structure (I) for synthesis. A key step in the route to these compounds (Li & Lowary, 2008) was a base-promoted ring contraction of epoxyketone (II) [see Fig. 1] (Hamon & Shirley, 1988), which gave a 1:1 mixture of two steroisomeric products (III) and (IV), both as racemic mixtures. In these products, it was critical to determine the relative configuration of the carbon bearing the OH group and the cyclopropane ring and doing so by spectroscopic methods was not possible. Therefore, derivatives of both (III) and (IV) were prepared in hopes of obtaining a crystalline material. We were pleased to discover that esterification of (IV) with 4-nitrobenzoyl chloride gave a crystalline product (V) from which the relative configuration of these two groups could be established by X-ray crystallography.

The molecular structure of (V) is shown in Fig. 2. In common with other bicyclo[3.1.0]hexane systems for which crystal structures have been reported (examples: Gurskaya *et al.*, 1990; Gurskaya *et al.*, 1996; Gallucci *et al.*, 2000; Garcia *et al.*, 1992; Guthrie *et al.*, 1981; Màrton-Merész *et al.*, 1983; Biswas *et al.*, 1996; Bai *et al.*, 2004), the five-membered ring is puckered into an envelope in which C3 is above the plane formed by C1, C2, C4 and C5. This places C3 *cis* to the cyclopropane moiety that is fused to the cyclopentane ring. As can clearly be seen, the relative configuration of the stereogenic centre substituted with the 4-nitrobenzoyloxy group and the cyclopropane is *trans*. Thus, it is possible to establish the structure of (IV) and, by inference, (III). The methoxymethoxy group present in the side chain adopted the expected conformation (Wolfe, 1972) in which the two O atoms are *gauche* to each other.

### Experimental

1-[2'-(methoxymethoxy)-1'-4-nitrobenzoyloxyethyl]bicyclo[3.1.0]hexan-4-one (V). To a stirred solution of (IV) (1.23 g, 6.15 mmol) in CH_2_Cl_2_-pyridine (10:1, 8.8 ml) was added 4-nitrobenzoyl chloride (1.36 g, 7.38 mmol) at 273 K. The mixture was then warmed to room temperature and stirred for 1 h. The reaction mixture was quenched by adding CH_3_OH, and then diluted with CH_2_Cl_2_. The solution was washed with 1 *M* HCl and water. The organic layer was dried (Na_2_SO_4_), filtered, concentrated, and the residue was purified by chromatography (1:1 EtOAc-hexane) to provide the product (V) as a light yellow solid (yield 1.60 g, 76%). This material was recrystallized from CH_2_Cl_2_ to provide a crystalline solid (m.p. 380–382 K). *R*_f_ 0.36 (1:1 EtOAc-Hexane); ^1^H NMR (500 MHz, CDCl_3_, δ_H_) 8.32–8.30 (m, 2 H, Ar), 8.23–8.21 (m, 2 H, Ar), 5.11 (dd, 1 H, J = 4.7, 7.0 Hz, H-7), 4.67–4.64 (m, 2 H, OCH_2_O), 3.95–3.88 (m, 2 H, MOMOCH_2_), 3.35 (s, 3 H, OCH_3_), 2.40–2.32 (m, 1 H, H-3), 2.16–2.13 (m, 3 H, H-2, H-3), 2.00 (dd, 1 H, J = 3.6, 9.4 Hz, H-5), 1.47 (dd, 1 H, J = 5.2, 9.4 Hz, H-6), 1.28 (dd, 1 H, J = 3.6, 5.2 Hz, H-6); ^13^C NMR (125 MHz, CDCl_3_, δ_C_) 212.3 (C-4), 163.9 (C-11), 150.7 (Ar), 135.2 (Ar), 130.4 (Ar *x* 2), 123.6 (Ar *x* 2), 96.6 (C-9), 76.2 (C-7), 67.3 (C-8), 55.5 (C-10), 34.3 (C-1), 32.8 (C-5), 32.6 (C-3), 22.7 (C-2), 17.9 (C-6). HRMS (ESI) m/*z* calculated for C_17_H_19_NO_7_ + Na: 372.1054, found: 372.1055.

### Refinement

Hydrogen atoms were generated in idealized positions (according to the *sp*^2^ or *sp*^3^ geometries of their parent carbon or oxygen atoms), and then refined using a riding model with fixed C—H distances (C—H = 0.95–1.00 Å) and with *U*_iso_(H) = 1.2*U*_eq_(C).

### Crystal data

**Table:**

C_17_H_19_NO_7_	*Z* = 2
*M**_r_* = 349.33	*F*_000_ = 368
Triclinic, *P*1	*D*_x_ = 1.381 Mg m^−^^3^
Hall symbol: -P 1	Mo *K*α radiation λ = 0.71073 Å
*a* = 8.3387 (5) Å	Cell parameters from 7818 reflections
*b* = 10.1389 (6) Å	θ = 2.6–27.5º
*c* = 10.4935 (6) Å	µ = 0.11 mm^−^^1^
α = 98.5259 (8)º	*T* = 193 (2) K
β = 100.4967 (8)º	Fragment, colourless
γ = 101.1562 (7)º	0.52 × 0.50 × 0.47 mm
*V* = 840.22 (9) Å^3^	

### Data collection

**Table:**

Bruker PLATFORM diffractometer/SMART 1000 CCD area-detector	3825 independent reflections
Radiation source: fine-focus sealed tube	3476 reflections with *I* > 2σ(*I*)
Monochromator: graphite	*R*_int_ = 0.009
Detector resolution: 8.192 pixels mm^-1^	θ_max_ = 27.5º
*T* = 193(2) K	θ_min_ = 2.0º
ω scans	*h* = −10→10
Absorption correction: multi-scan(SADABS; Sheldrick, 2003)	*k* = −13→13
*T*_min_ = 0.946, *T*_max_ = 0.951	*l* = −13→13
7436 measured reflections	

### Refinement

**Table:**

Refinement on *F*^2^	Secondary atom site location: difference Fourier map
Least-squares matrix: full	Hydrogen site location: inferred from neighbouring sites
*R*[*F*^2^ > 2σ(*F*^2^)] = 0.042	H-atom parameters constrained
*wR*(*F*^2^) = 0.120	*w* = 1/[σ^2^(*F*_o_^2^) + (0.0703*P*)^2^ + 0.1568*P*] where *P* = (*F*_o_^2^ + 2*F*_c_^2^)/3
*S* = 1.05	(Δ/σ)_max_ = 0.013
3825 reflections	Δρ_max_ = 0.31 e Å^−^^3^
227 parameters	Δρ_min_ = −0.17 e Å^−^^3^
Primary atom site location: structure-invariant direct methods	Extinction correction: none

### Special details

**Table:**

Geometry. All e.s.d.'s (except the e.s.d. in the dihedral angle between two l.s. planes) are estimated using the full covariance matrix. The cell e.s.d.'s are taken into account individually in the estimation of e.s.d.'s in distances, angles and torsion angles; correlations between e.s.d.'s in cell parameters are only used when they are defined by crystal symmetry. An approximate (isotropic) treatment of cell e.s.d.'s is used for estimating e.s.d.'s involving l.s. planes.
Refinement. Refinement of *F*^2^ against ALL reflections. The weighted *R*-factor *wR* and goodness of fit *S* are based on *F*^2^, conventional *R*-factors *R* are based on *F*, with *F* set to zero for negative *F*^2^. The threshold expression of *F*^2^ > σ(*F*^2^) is used only for calculating *R*-factors(gt) *etc*. and is not relevant to the choice of reflections for refinement. *R*-factors based on *F*^2^ are statistically about twice as large as those based on *F*, and *R*- factors based on ALL data will be even larger.

### Fractional atomic coordinates and isotropic or equivalent isotropic displacement parameters (Å^2^)

**Table:**

	*x*	*y*	*z*	*U*_iso_*/*U*_eq_	
O1	0.09546 (12)	0.07242 (11)	0.73892 (11)	0.0553 (3)	
O2	0.27720 (9)	0.22849 (8)	0.35099 (8)	0.03307 (19)	
O3	0.36653 (10)	0.48159 (9)	0.28812 (8)	0.0367 (2)	
O4	0.19060 (11)	0.53166 (10)	0.10951 (9)	0.0469 (2)	
O5	0.54644 (13)	0.22855 (12)	0.42875 (11)	0.0650 (3)	
O6	0.43439 (13)	−0.32377 (10)	−0.08313 (10)	0.0534 (3)	
O7	0.18678 (14)	−0.29604 (11)	−0.15763 (10)	0.0602 (3)	
N	0.32018 (14)	−0.26560 (10)	−0.07714 (10)	0.0398 (2)	
C1	0.16536 (13)	0.31828 (10)	0.53155 (10)	0.0302 (2)	
C2	−0.01745 (14)	0.24464 (13)	0.47296 (12)	0.0384 (3)	
H2A	−0.0267	0.1788	0.3908	0.046*	
H2B	−0.0864	0.3112	0.4527	0.046*	
C3	−0.07499 (15)	0.16936 (15)	0.57953 (14)	0.0458 (3)	
H3A	−0.1392	0.2220	0.6298	0.055*	
H3B	−0.1466	0.0775	0.5389	0.055*	
C4	0.08438 (15)	0.15744 (12)	0.66935 (12)	0.0391 (3)	
C5	0.22604 (14)	0.26104 (11)	0.65309 (11)	0.0344 (2)	
H5	0.3421	0.2453	0.6717	0.041*	
C6	0.19962 (16)	0.40686 (12)	0.66433 (11)	0.0379 (3)	
H6A	0.1024	0.4264	0.6994	0.046*	
H6B	0.3003	0.4831	0.6890	0.046*	
C7	0.28480 (13)	0.35255 (10)	0.44415 (10)	0.0294 (2)	
H7	0.4011	0.3873	0.4990	0.035*	
C8	0.24071 (13)	0.45645 (11)	0.36257 (11)	0.0331 (2)	
H8A	0.1291	0.4203	0.3028	0.040*	
H8B	0.2384	0.5422	0.4206	0.040*	
C9	0.34064 (15)	0.57459 (13)	0.20414 (12)	0.0414 (3)	
H9A	0.3415	0.6636	0.2582	0.050*	
H9B	0.4348	0.5892	0.1586	0.050*	
C10	0.18474 (19)	0.41015 (17)	0.01905 (14)	0.0547 (4)	
H10A	0.0777	0.3849	−0.0454	0.066*	
H10B	0.1956	0.3356	0.0674	0.066*	
H10C	0.2769	0.4264	−0.0270	0.066*	
C11	0.41618 (14)	0.18250 (11)	0.35071 (11)	0.0332 (2)	
C12	0.38783 (13)	0.06385 (10)	0.23884 (10)	0.0297 (2)	
C13	0.52157 (14)	0.00427 (11)	0.22180 (11)	0.0328 (2)	
H13	0.6274	0.0381	0.2811	0.039*	
C14	0.50030 (14)	−0.10469 (11)	0.11803 (11)	0.0328 (2)	
H14	0.5905	−0.1461	0.1051	0.039*	
C15	0.34449 (14)	−0.15100 (10)	0.03447 (10)	0.0317 (2)	
C16	0.20974 (15)	−0.09435 (12)	0.04924 (12)	0.0385 (3)	
H16	0.1041	−0.1290	−0.0101	0.046*	
C17	0.23241 (14)	0.01446 (12)	0.15285 (12)	0.0361 (2)	
H17	0.1416	0.0553	0.1651	0.043*	

### Atomic displacement parameters (Å^2^)

**Table:**

	*U*^11^	*U*^22^	*U*^33^	*U*^12^	*U*^13^	*U*^23^
O1	0.0480 (5)	0.0566 (6)	0.0701 (7)	0.0130 (4)	0.0196 (5)	0.0295 (5)
O2	0.0283 (4)	0.0314 (4)	0.0349 (4)	0.0070 (3)	0.0051 (3)	−0.0062 (3)
O3	0.0310 (4)	0.0466 (5)	0.0339 (4)	0.0100 (3)	0.0069 (3)	0.0108 (3)
O4	0.0386 (5)	0.0592 (6)	0.0436 (5)	0.0144 (4)	0.0021 (4)	0.0154 (4)
O5	0.0436 (5)	0.0701 (7)	0.0627 (6)	0.0286 (5)	−0.0171 (5)	−0.0310 (5)
O6	0.0586 (6)	0.0459 (5)	0.0544 (6)	0.0203 (4)	0.0154 (5)	−0.0091 (4)
O7	0.0642 (7)	0.0551 (6)	0.0463 (5)	0.0174 (5)	−0.0092 (5)	−0.0168 (4)
N	0.0500 (6)	0.0325 (5)	0.0347 (5)	0.0094 (4)	0.0092 (4)	−0.0001 (4)
C1	0.0295 (5)	0.0306 (5)	0.0287 (5)	0.0084 (4)	0.0044 (4)	0.0006 (4)
C2	0.0277 (5)	0.0490 (6)	0.0357 (6)	0.0080 (5)	0.0045 (4)	0.0033 (5)
C3	0.0306 (6)	0.0577 (8)	0.0489 (7)	0.0069 (5)	0.0108 (5)	0.0110 (6)
C4	0.0372 (6)	0.0409 (6)	0.0414 (6)	0.0102 (5)	0.0132 (5)	0.0075 (5)
C5	0.0317 (5)	0.0365 (5)	0.0335 (5)	0.0081 (4)	0.0040 (4)	0.0052 (4)
C6	0.0446 (6)	0.0356 (6)	0.0311 (5)	0.0095 (5)	0.0081 (5)	−0.0018 (4)
C7	0.0277 (5)	0.0277 (5)	0.0292 (5)	0.0063 (4)	0.0042 (4)	−0.0033 (4)
C8	0.0310 (5)	0.0350 (5)	0.0332 (5)	0.0091 (4)	0.0075 (4)	0.0035 (4)
C9	0.0384 (6)	0.0440 (6)	0.0402 (6)	0.0055 (5)	0.0052 (5)	0.0126 (5)
C10	0.0476 (7)	0.0665 (9)	0.0422 (7)	0.0082 (6)	−0.0013 (6)	0.0063 (6)
C11	0.0316 (5)	0.0335 (5)	0.0326 (5)	0.0104 (4)	0.0040 (4)	0.0004 (4)
C12	0.0307 (5)	0.0282 (5)	0.0294 (5)	0.0071 (4)	0.0065 (4)	0.0028 (4)
C13	0.0304 (5)	0.0332 (5)	0.0332 (5)	0.0096 (4)	0.0033 (4)	0.0024 (4)
C14	0.0354 (5)	0.0314 (5)	0.0345 (5)	0.0128 (4)	0.0099 (4)	0.0057 (4)
C15	0.0400 (6)	0.0260 (5)	0.0285 (5)	0.0068 (4)	0.0089 (4)	0.0025 (4)
C16	0.0314 (5)	0.0375 (6)	0.0395 (6)	0.0054 (4)	0.0012 (4)	−0.0040 (5)
C17	0.0292 (5)	0.0366 (5)	0.0397 (6)	0.0095 (4)	0.0057 (4)	−0.0018 (4)

### Geometric parameters (Å, °)

**Table:**

O1—C4	1.2155 (16)	C5—H5	1.0000
O2—C11	1.3306 (13)	C6—H6A	0.9900
O2—C7	1.4573 (11)	C6—H6B	0.9900
O3—C9	1.4029 (14)	C7—C8	1.5100 (15)
O3—C8	1.4249 (13)	C7—H7	1.0000
O4—C9	1.3962 (14)	C8—H8A	0.9900
O4—C10	1.4262 (18)	C8—H8B	0.9900
O5—C11	1.1978 (14)	C9—H9A	0.9900
O6—N	1.2194 (14)	C9—H9B	0.9900
O7—N	1.2222 (14)	C10—H10A	0.9800
N—C15	1.4771 (13)	C10—H10B	0.9800
C1—C6	1.4880 (14)	C10—H10C	0.9800
C1—C7	1.4991 (15)	C11—C12	1.5005 (14)
C1—C5	1.5223 (15)	C12—C17	1.3905 (15)
C1—C2	1.5301 (15)	C12—C13	1.3936 (15)
C2—C3	1.5395 (18)	C13—C14	1.3909 (15)
C2—H2A	0.9900	C13—H13	0.9500
C2—H2B	0.9900	C14—C15	1.3799 (15)
C3—C4	1.5194 (17)	C14—H14	0.9500
C3—H3A	0.9900	C15—C16	1.3796 (16)
C3—H3B	0.9900	C16—C17	1.3870 (15)
C4—C5	1.4746 (16)	C16—H16	0.9500
C5—C6	1.5271 (16)	C17—H17	0.9500
			
C11—O2—C7	118.68 (8)	O2—C7—H7	109.6
C9—O3—C8	113.65 (9)	C1—C7—H7	109.6
C9—O4—C10	112.74 (10)	C8—C7—H7	109.6
O6—N—O7	124.19 (10)	O3—C8—C7	106.79 (8)
O6—N—C15	117.96 (10)	O3—C8—H8A	110.4
O7—N—C15	117.85 (10)	C7—C8—H8A	110.4
C6—C1—C7	117.45 (9)	O3—C8—H8B	110.4
C6—C1—C5	60.96 (7)	C7—C8—H8B	110.4
C7—C1—C5	118.28 (9)	H8A—C8—H8B	108.6
C6—C1—C2	116.10 (9)	O4—C9—O3	113.68 (10)
C7—C1—C2	120.97 (9)	O4—C9—H9A	108.8
C5—C1—C2	108.03 (9)	O3—C9—H9A	108.8
C1—C2—C3	105.52 (9)	O4—C9—H9B	108.8
C1—C2—H2A	110.6	O3—C9—H9B	108.8
C3—C2—H2A	110.6	H9A—C9—H9B	107.7
C1—C2—H2B	110.6	O4—C10—H10A	109.5
C3—C2—H2B	110.6	O4—C10—H10B	109.5
H2A—C2—H2B	108.8	H10A—C10—H10B	109.5
C4—C3—C2	105.68 (9)	O4—C10—H10C	109.5
C4—C3—H3A	110.6	H10A—C10—H10C	109.5
C2—C3—H3A	110.6	H10B—C10—H10C	109.5
C4—C3—H3B	110.6	O5—C11—O2	125.11 (10)
C2—C3—H3B	110.6	O5—C11—C12	124.22 (10)
H3A—C3—H3B	108.7	O2—C11—C12	110.68 (9)
O1—C4—C5	125.17 (11)	C17—C12—C13	120.31 (10)
O1—C4—C3	125.78 (11)	C17—C12—C11	121.15 (9)
C5—C4—C3	108.97 (10)	C13—C12—C11	118.53 (9)
C4—C5—C1	107.41 (9)	C14—C13—C12	120.05 (10)
C4—C5—C6	115.41 (10)	C14—C13—H13	120.0
C1—C5—C6	58.41 (7)	C12—C13—H13	120.0
C4—C5—H5	119.9	C15—C14—C13	118.12 (10)
C1—C5—H5	119.9	C15—C14—H14	120.9
C6—C5—H5	119.9	C13—C14—H14	120.9
C1—C6—C5	60.63 (7)	C16—C15—C14	123.09 (10)
C1—C6—H6A	117.7	C16—C15—N	118.07 (10)
C5—C6—H6A	117.7	C14—C15—N	118.84 (10)
C1—C6—H6B	117.7	C15—C16—C17	118.32 (10)
C5—C6—H6B	117.7	C15—C16—H16	120.8
H6A—C6—H6B	114.8	C17—C16—H16	120.8
O2—C7—C1	108.39 (8)	C16—C17—C12	120.11 (10)
O2—C7—C8	106.47 (8)	C16—C17—H17	119.9
C1—C7—C8	113.09 (8)	C12—C17—H17	119.9
			
C6—C1—C2—C3	−52.63 (13)	C9—O3—C8—C7	178.58 (9)
C7—C1—C2—C3	154.16 (10)	O2—C7—C8—O3	−62.70 (10)
C5—C1—C2—C3	13.23 (12)	C1—C7—C8—O3	178.37 (8)
C1—C2—C3—C4	−20.30 (13)	C10—O4—C9—O3	−63.98 (14)
C2—C3—C4—O1	−156.31 (13)	C8—O3—C9—O4	−60.59 (13)
C2—C3—C4—C5	20.54 (14)	C7—O2—C11—O5	6.45 (18)
O1—C4—C5—C1	164.54 (12)	C7—O2—C11—C12	−173.72 (8)
C3—C4—C5—C1	−12.34 (13)	O5—C11—C12—C17	179.13 (13)
O1—C4—C5—C6	−132.77 (13)	O2—C11—C12—C17	−0.71 (15)
C3—C4—C5—C6	50.36 (13)	O5—C11—C12—C13	−1.43 (19)
C6—C1—C5—C4	109.58 (11)	O2—C11—C12—C13	178.73 (9)
C7—C1—C5—C4	−142.96 (10)	C17—C12—C13—C14	0.32 (17)
C2—C1—C5—C4	−0.81 (12)	C11—C12—C13—C14	−179.12 (10)
C7—C1—C5—C6	107.46 (10)	C12—C13—C14—C15	−0.23 (16)
C2—C1—C5—C6	−110.39 (10)	C13—C14—C15—C16	−0.01 (17)
C7—C1—C6—C5	−108.79 (10)	C13—C14—C15—N	179.31 (10)
C2—C1—C6—C5	97.02 (11)	O6—N—C15—C16	−173.37 (11)
C4—C5—C6—C1	−95.56 (11)	O7—N—C15—C16	6.64 (16)
C11—O2—C7—C1	−120.22 (10)	O6—N—C15—C14	7.28 (16)
C11—O2—C7—C8	117.82 (10)	O7—N—C15—C14	−172.71 (11)
C6—C1—C7—O2	155.15 (9)	C14—C15—C16—C17	0.16 (18)
C5—C1—C7—O2	85.13 (11)	N—C15—C16—C17	−179.17 (10)
C2—C1—C7—O2	−51.99 (12)	C15—C16—C17—C12	−0.07 (18)
C6—C1—C7—C8	−87.05 (11)	C13—C12—C17—C16	−0.17 (18)
C5—C1—C7—C8	−157.06 (9)	C11—C12—C17—C16	179.26 (11)
C2—C1—C7—C8	65.82 (12)		
